# Gene Dispensability in Escherichia coli Grown in Thirty Different Carbon Environments

**DOI:** 10.1128/mBio.02259-20

**Published:** 2020-09-29

**Authors:** Madeline Tong, Shawn French, Sara S. El Zahed, Wai kit Ong, Peter D. Karp, Eric D. Brown

**Affiliations:** a Michael G. DeGroote Institute for Infectious Disease Research, Department of Biochemistry and Biomedical Sciences, McMaster University, Hamilton, Ontario, Canada; b Bioinformatics Research Group, SRI International, Menlo Park, California, USA; Georgia Institute of Technology School of Biological Sciences

**Keywords:** *Escherichia coli*, carbon metabolism, carbon source, central metabolism, genetics

## Abstract

Central metabolism is a topic that has been studied for decades, and yet, this process is still not fully understood in Escherichia coli, perhaps the most amenable and well-studied model organism in biology. To further our understanding, we used a high-throughput method to measure the growth kinetics of each of 3,796 E. coli single-gene deletion mutants in 30 different carbon sources. In total, there were 342 genes (9.01%) encompassing a breadth of biological functions that showed a growth phenotype on at least 1 carbon source, demonstrating that carbon metabolism is closely linked to a large number of processes in the cell. We identified 74 genes that showed low growth in 90% of conditions, defining a set of genes which are essential in nutrient-limited media, regardless of the carbon source. The data are compiled into a Web application, Carbon Phenotype Explorer (CarPE), to facilitate easy visualization of growth curves for each mutant strain in each carbon source. Our experimental data matched closely with the predictions from the EcoCyc metabolic model which uses flux balance analysis to predict growth phenotypes. From our comparisons to the model, we found that, unexpectedly, phosphoenolpyruvate carboxylase (*ppc*) was required for robust growth in most carbon sources other than most trichloroacetic acid (TCA) cycle intermediates. We also identified 51 poorly annotated genes that showed a low growth phenotype in at least 1 carbon source, which allowed us to form hypotheses about the functions of these genes. From this list, we further characterized the *ydhC* gene and demonstrated its role in adenosine efflux.

## Abstract (precis)

While there has been much study of bacterial gene dispensability, there is a lack of comprehensive genome-scale examinations of the impact of gene deletion on growth in different carbon sources. In this context, a lot can be learned from such experiments in the model microbe Escherichia coli where much is already understood and there are existing tools for the investigation of carbon metabolism and physiology (1). Gene deletion studies have practical potential in the field of antibiotic drug discovery where there is emerging interest in bacterial central metabolism as a target for new antibiotics (2). Furthermore, some carbon utilization pathways have been shown to be critical for initiating and maintaining infection for certain pathogens and sites of infection (3–5). Here, with the use of high-throughput solid medium phenotyping methods, we have generated kinetic growth measurements for 3,796 genes under 30 different carbon source conditions. This data set provides a foundation for research that will improve our understanding of genes with unknown function, aid in predicting potential antibiotic targets, validate and advance metabolic models, and help to develop our understanding of E. coli metabolism.

## INTRODUCTION

Central metabolism is an essential process that generates all of the energy and biosynthetic precursors required for bacterial survival. It is a highly regulated network of pathways that respond to extracellular concentrations of carbon and nutrients (6). Escherichia coli has a robust regulatory framework that allows it to best use the nutrients and carbon source available in its environment (7). This complex regulatory network has multiple levels of control mechanisms, including changes in gene expression through transcription factors and modulations in enzyme activity with allosteric inhibition or posttranslational modifications (8). For example, transcription factors, such as the cAMP receptor protein, regulate the usage of secondary carbon sources through a process called carbon catabolite repression (9). Carbon catabolite repression is thought to control the expression of as much as 10% of the genome (10). Using this process, E. coli genes are differentially regulated based on the growth medium, where specific genes become essential for bacterial survival. For example, the E. coli genome contains about 4,300 genes, with 303 being essential for growth in the rich microbiological growth medium LB (11, 12). However, gene essentiality is contextual and dependent on the growth condition (13). In nutrient-limited media containing glucose or glycerol as a carbon source, an additional 119 genes become essential (11, 14).

The E. coli genome has been well studied; however, around 35% of its genes are still poorly characterized (1, 15). Identifying experimental phenotypes in gene knockout mutants is a powerful way to understand functions of specific gene products (16). For poorly annotated genes in the E. coli genome, such phenotypes can be used to hypothesize the gene’s function. Indeed, the development of high-throughput gene inactivation techniques has led to the generation of an E. coli mutant collection (Keio collection) which facilitates genome-wide studies of phenotypes (11, 17, 18). Previous studies have successfully used systematic analyses of phenotypes to gain an understanding of genetic interactions and to characterize genes with unknown functions. (16, 19–21).

To generate a large data set of phenotypes, researchers have developed high-throughput experimental approaches. For example, phenotype microarrays (PMAs) can be used to evaluate the growth fitness of specific strains under hundreds of conditions (22, 23). Although PMAs have been applied to 1,400 strains from the Keio collection, it is a resource-intensive process that is difficult to apply to larger libraries (24). Transposon mutagenesis and sequencing (TnSeq) studies have also been used to probe gene essentiality efficiently using one-pot selection approaches for a large number of conditions (19). While these studies generate a growth fitness score for each gene by monitoring the abundance of all mutants, they cannot generate individual kinetic measurements for each mutant. Additionally, the pooling of many mutants into a single culture has the risk of creating competition between strains, making it difficult to assess the growth of a slow-growing strain across multiple conditions (25). Likewise, chemical complementation between strains can be confounding in these pooled approaches due to the provision of biosynthetic intermediates from one mutant to another (26). Pioneered first by those studying yeast genetics, arraying colonies on solid media has become a popular high-throughput method for large-scale investigations of growth phenotypes (16, 27–29). This approach can be adjusted to high-throughput measurements of growth fitness based on colony sizes of each strain. Such screens typically generate a growth fitness score for each strain. This information has successfully been used to understand gene function and chromosome organization and to provide insight to the mechanism of certain drugs (16, 27, 30).

Many studies have successfully used the E. coli gene knockout collection (Keio collection) to study metabolism (14, 20, 31). Phenotypic screens of this collection have studied a few carbon sources to date where the focus has been on validating metabolic models (14, 16, 32). E. coli has the remarkable ability to use many carbon sources for growth which makes it possible to exhaustively probe central metabolism pathways using different carbon sources. Since methodologies differ from lab to lab, unifying different gene essentiality data sets has been difficult (33). This speaks to the need for more comprehensive data sets that explore a large number of conditions using the same strains and methodology.

Here, we expand on previous research and study the growth kinetics of each gene deletion mutant in the Keio collection, by growing the mutants on 30 different carbon sources and probing comprehensively the pathways of central metabolism in E. coli. Using a chemically defined minimal medium (morpholinepropanesulfonic acid [MOPS]) and changing only the carbon source (34), we collected 24-hour growth kinetics for each deletion mutant growing on solid agar at high density, i.e., 1,536 colonies per plate. We compared these experimental data against the EcoCyc metabolic model that uses flux balance analysis to predict growth phenotypes and identified discrepancies which can help improve the accuracy of these models. This data set presents an opportunity to investigate the gaps in knowledge of bacterial metabolism and physiology. Our focus was on a lab strain of E. coli (BW25113), a representative K-12 strain where extensive experimental, genome curation, and metabolic modeling efforts could provide ample context for our investigations. Central metabolism is highly conserved, even between different domains of life (35, 36). Curated databases of metabolic pathways have been successfully used to predict metabolic networks of different organisms simply based on genome sequence, and therefore, advancing our knowledge on a simple model microbe has profound implications for understanding metabolism as a whole (37). Here, we have identified trends in how the E. coli growth rate changes with different carbon sources. Using this data set, we investigated the function of the enigmatic *ydhC* gene and implicated it in adenosine efflux. Finally, we developed a Web application which can be easily used for the visualization of growth curves for each mutant strain in each carbon source, allowing for a straightforward analysis of the data set without programming expertise.

## RESULTS

### A genome-wide screen of E. coli in different carbon sources.

**FIG 1:**
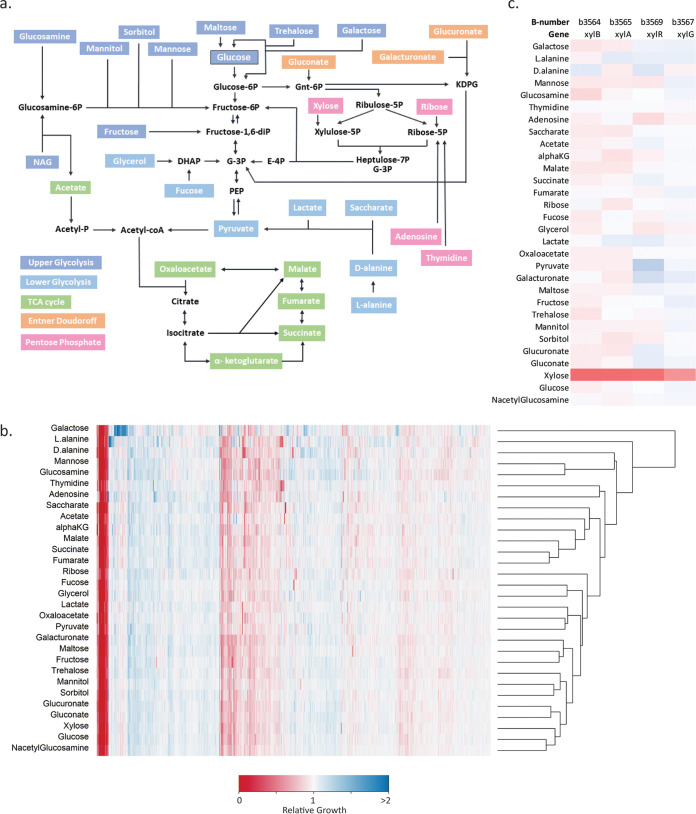
Summary of data collected in this study. (a) Carbon sources tested in this study. Carbon sources can enter central metabolism through different pathways. They were sorted into four different categories based on the metabolite to which it would be metabolized. NAG, *N*-acetylglucosamine; P, phosphate; DHAP, dihydroxyacetone phosphate; G-3P, glyceraldehyde-3-phosphate; E-4P, erythrose-4-phosphate; Gnt-6P, gluconate-6-phosphate; KDPG, 2-keto-3-deoxy-6-phosphogluconate. (b) Heatmap visualization of the normalized growth at 24 h of each gene in every carbon source. Each line on the *x* axis represents the growth of a gene deletion mutant. The *y* axis shows the carbon source in which the mutant was grown. Red means lower growth than the expected growth and blue means higher growth than expected. (c) An example of a group of genes involved in xylose metabolism that clustered in the heatmap shown in b. Genes *xylB*, *A*, *R*, and *G* encode proteins involved in xylose transport, regulation, and metabolism and showed growth defects, specifically on xylose.

We first elected to do a broad survey of the carbon sources that E. coli is able to utilize for growth using the Biolog phenotype microarray (22). This commercially available platform contains 190 different carbon sources and can be used for the rapid identification of carbon sources for bacterial growth. We identified 74 carbon sources that could support the growth of our lab strain of E. coli BW25113 in MOPS minimal media (see Fig. S1 in the supplemental material). Based on these results, we narrowed the list to 30 carbon sources that feed different pathways of central metabolism, which are highlighted in Fig. 1a. We categorized each carbon source according to the pathway intermediate that E. coli metabolizes it to. The sugars which are catabolized to intermediates in the glycolysis pathway prior to glyceraldehyde-3-phosphate (G-3P) are categorized as upper glycolysis. Lower glycolysis carbon sources are metabolized to G-3P or pyruvate. Tricarboxylic acid (TCA) cycle carbon sources are intermediates within that pathway. Entner-Doudoroff carbon sources are metabolized through the metabolite 2-keto-deoxy-6-phosphogluconate (KDPG). Finally, pentose phosphate compounds are catabolized to metabolic intermediates in the pentose phosphate pathway (Fig. 1a).

10.1128/mBio.02259-20.1 FIG S1 The biology phenotype microarray was performed on E. coli BW25113 using MOPS minimal media. Purple wells indicate a reaction with the tetrazolium dye and show cellular respiration. These wells contain carbon sources that can be used by this strain of E. coli. Download FIG S1, TIF file, 0.4 MB. Copyright © 2020 Tong et al. 2020 Tong et al. This content is distributed under the terms of the Creative Commons Attribution 4.0 International license.

To evaluate the impact of each gene deletion on the growth of E. coli in each carbon source, we screened the Keio collection, an ordered library of single-gene knockouts (11), in solid minimal media containing each carbon source. We completed this evaluation by arraying each mutant of the collection onto solid agar media in duplicate and monitoring the growth of each colony over the course of 24 hours (see Fig. S2 in the supplemental material). The final 24-h endpoint growth was normalized using a method described by French et al. (27) and produced high-resolution colony arrays. We normalized each carbon source treatment individually, given the unique growth characteristics of E. coli observed in each carbon environment. These endpoint biomass values formed a highly replicating final data set of growth amplitudes for 3,796 genes tested across 30 different carbon sources (see Fig. S3 and Table S1A in the supplemental material). Indeed, the endpoint values were of great utility for understanding the trends in gene essentiality. The kinetic data, consisting of 72 growth measurements over 24 h, were used to verify these growth patterns since the growth curve of E. coli differed depending on the carbon source.

10.1128/mBio.02259-20.2 FIG S2 Workflow diagram describing the process of screening the E. coli single-gene knockout collection. Using the Singer Rotor, plates containing the library were first grown on LB agar. Then, they were pinned onto a minimal media plate and grown for 24 h to deplete any nutrients that may have been carried over. These plates were then used to inoculate two new assay plates as technical replicates. Plates were scanned every 20 min to obtain a total of 73 images of the same plate at different time points over the course of 24 hours. Plates were analyzed using ImageJ in order to determine the integrated density (approximates the amount of bacteria) of each colony at each time point. Download FIG S2, TIF file, 0.2 MB. Copyright © 2020 Tong et al. 2020 Tong et al. This content is distributed under the terms of the Creative Commons Attribution 4.0 International license.

10.1128/mBio.02259-20.3 FIG S3 (a) A scatterplot comparing the normalized integrated density, a value based on the final colony size relative to the rest of the condition, between two technical replicates. A correlation coefficient of 0.911 demonstrates highly reproducible results. (b) Histogram showing the distribution of all the normalized growth values based on the final colony sizes in our data set. The red dotted line shows the cutoff of 3 standard deviations from the mean. Some 342 mutants showed growth below that cutoff in any carbon source. (c) We grew two plates of WT E. coli on solid media pinned in 1,534-colony-density in 5 different carbon sources, namely, fumarate, glucose, pyruvate, sorbitol, and xylose. The black line corresponds to the average growth curve of the WT E. coli, and the red line represents the growth curve of the unperturbed Keio mutants. Download FIG S3, TIF file, 0.1 MB. Copyright © 2020 Tong et al. 2020 Tong et al. This content is distributed under the terms of the Creative Commons Attribution 4.0 International license.

10.1128/mBio.02259-20.5 TABLE S1 (A) Endpoint biomass dataset. (B) Comparison to previous MOPS glucose dataset. Download Table S1, XLSX file, 1.7 MB. Copyright © 2020 Tong et al. 2020 Tong et al. This content is distributed under the terms of the Creative Commons Attribution 4.0 International license.

We subjected our results to two-dimensional hierarchical clustering as a check on consistency of the data with known metabolism (Fig. 1b). This produced a heat map where carbon sources (*y* axis) entering central metabolism in similar pathways clustered, while on the *x* axis, genes in similar pathways clustered. For example, the carbon sources adenosine and thymidine are both nucleosides that clustered based on their Keio collection genetic responses. TCA cycle intermediates α-ketoglutarate, malate, succinate, and fumarate clustered. On the *x* axis, genes belonging to the same operon with similar biological function or subunits of the same protein also clustered closely. Genes involved in thiamine biosynthesis (*thiHSCIFE*), despite being low growth under most conditions, clustered closely. As expected, *xylBARG* clustered closely, as they are all required specifically for growth on xylose (Fig. 1c). In E. coli, xylose is transported by the xylose ABC transporter. XylG is the ATP binding subunit of the transporter, while xylHF encodes the membrane subunit and periplasmic binding protein. As found in previous studies, we noticed that Δ*xylH* or Δ*xylF* single deletions were unable to prevent growth on xylose (38). Once in the cell, the sugar is isomerized by XylA into xylulose. XylB is a xylulokinase which converts xylulose into xylulose 5-phosphate where it can be further metabolized through the pentose phosphate pathway. XylR is a transcriptional activator that binds to the *xyl* promoters and is required for the expression of *xylAB* (38). Interestingly, galactose clustered outside all the other carbon sources because of gene deletions that improved growth on that substrate. In particular, this included *galS* and *galR*, which are both DNA binding repressors of the *gal* regulon genes (39). These genes encode regulatory enzymes that repress the expression of genes involved in galactose transport and metabolism pathways unless galactose is present (39). Together, hierarchical clustering showed trends that we would expect from our data based on prior knowledge in E. coli metabolism, indicating that our data set was biologically meaningful. Genes that are known to be in the same pathways showed a similar phenotypic pattern of growth across carbon sources.

### Genes important for E. coli carbon metabolism.

**FIG 2:**
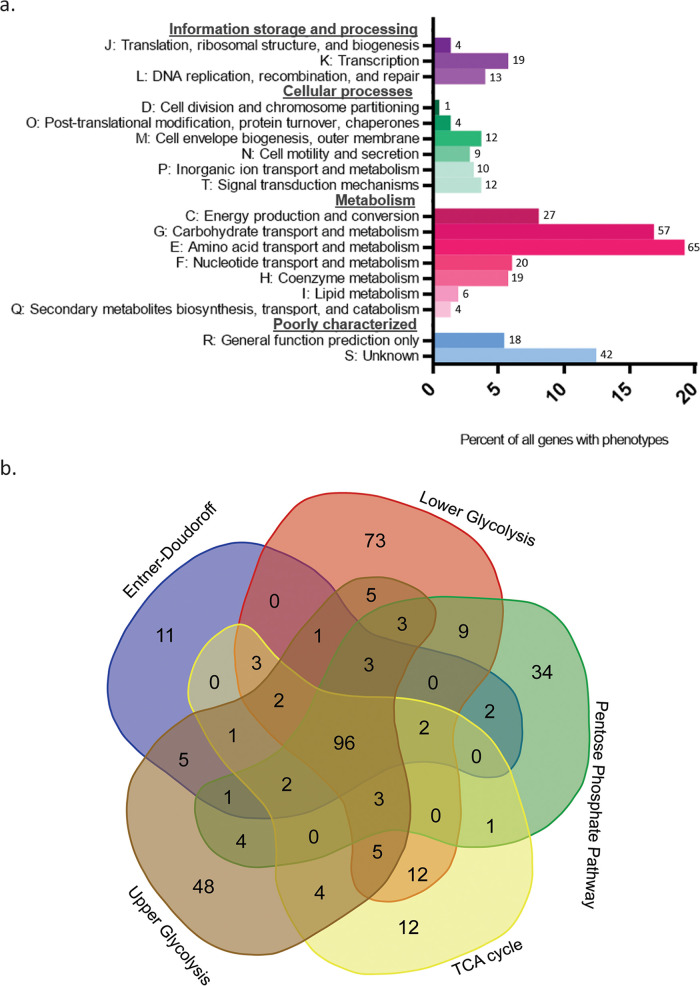
Analysis of the gene deletions leading to growth defects. (a) Functional analysis of genes with growth defects in any carbon source. Each gene was categorized into a single Cluster of Orthologous Groups (COG) (78) category. COG categories were based on the designations given in the current database, and genes without a category were manually curated based on current known functions. The genes in the R category are those that have predicted functions from homology or from experimental study. In the S category are genes that do not have any predicted functions. The bars represent the percentage of genes that belong in that category, while the numbers show the number of genes in that category. (b) The Venn diagram assigns the 342 genes required for growth in any carbon source to the five pathways shown. Each carbon source was categorized according to that described in Fig. 1a. Genes in a specific category means they were required for growth by at least one carbon source in that pathway. The names of the genes contained in each of the overlapping regions are specified in Table S3.

We were especially interested in identifying genes in E. coli required for robust growth on different carbon sources. Deletion mutants that resulted in a growth defect when grown in a specific carbon source imply that the function of the deleted gene is important for growth under that condition. To determine which gene deletions caused a low growth phenotype, we used a 3 standard deviation cutoff below the interquartile mean of our entire data set (40) (Fig. S3). On average, we identified 102 genes per carbon source that showed a growth defect, a number similar in scale to the 119 genes that were found to have an indispensable phenotype for growth in minimal media containing glucose or glycerol (Table 1) (11, 14). We note here that, while our experiments were conducted in solid media, this glucose data set had a 98% agreement with the results of a previous study of the Keio collection grown in liquid cultures of MOPS glucose (Table S1B) (11). In total, there were 342 genes (9.01%) showing a low growth phenotype on at least one carbon source (see Table S2A in the supplemental material). These mutants that showed low growth were largely enriched for genes involved in carbohydrate, amino acid, coenzyme, and nucleotide transport and metabolism. This is expected from a screen done in minimal media, which requires bacteria to synthesize their own amino acids, nucleotides, and vitamins (Fig. 2a). We also identified 51 genes with poorly annotated functions that showed a low growth phenotype in at least 1 carbon source (Table S2B). Finally, we identified 74 genes that showed low growth in 90% of conditions, defining a set of genes which are essential in all minimal media, regardless of the carbon source (Table S2C). It is interesting to note that while most of the genes with phenotypes are involved in metabolism, there were many genes that fell into different categories unrelated to metabolism. In all, our analysis demonstrated that carbon metabolism is closely linked to a large number of cellular processes.

**TABLE 1:** Summary statistics of data set

Parameter	Value
Total no. of genes tested	3,796
Avg hits^*a*^ per carbon source	102
Hits^*a*^ in at least one carbon source	342
Hits^*a*^ in at least 90% of carbon sources tested	74
Poorly annotated genes that are a hit^*a*^ in at least one condition	51

a Hits are defined as gene deletion mutants that had a final growth 3 SDs below the average growth.

10.1128/mBio.02259-20.6 TABLE S2 (A) A total of 342 genes with at least 1 phenotype. (B) A total of 74 genes with low growth in 90% of conditions. (C) A total of 51 poorly annotated genes with phenotypes. Download Table S2, XLSX file, 0.02 MB. Copyright © 2020 Tong et al. 2020 Tong et al. This content is distributed under the terms of the Creative Commons Attribution 4.0 International license.

10.1128/mBio.02259-20.7 TABLE S3 Genes in Venn diagram intersections (Fig. 2b). Download Table S3, XLSX file, 0.01 MB. Copyright © 2020 Tong et al. 2020 Tong et al. This content is distributed under the terms of the Creative Commons Attribution 4.0 International license.

To explore the functional genomics of carbon metabolism further, we compared the genes required for growth across the five different categories of carbon sources used in this study. For each of the categories described in Fig. 1a, we generated a list of genes that showed a low growth phenotype when deleted in at least one carbon source in that group. We compared these gene lists with a Venn diagram analysis to compare which genes had growth phenotypes that were unique to certain carbon pathways and which were shared with multiple pathways (Fig. 2b). As expected, genes encoding enzymes required to degrade a specific carbon source in a pathway showed specificity for that category. For example, the genes *edd*, *eda*, *uxaABCR*, and *idnK* are all required to metabolize gluconate, glucoronate, and galacturonate and were all specific to the Entner-Doudoroff category (see Table S3 in the supplemental material). Overall, 96 genes showed a low growth phenotype in at least 1 carbon source in each category (Table S3). These genes are not specific to carbon degradation pathways in central metabolism and are instead specific to the nutrient biosynthetic pathways required in nutrient-limited media. It is interesting to note that lower glycolysis carbon sources have significant overlap with the TCA cycle category, as these carbon sources are closely linked in central metabolism.

### The growth rate of E. coli varies depending on carbon source.

**FIG 3:**
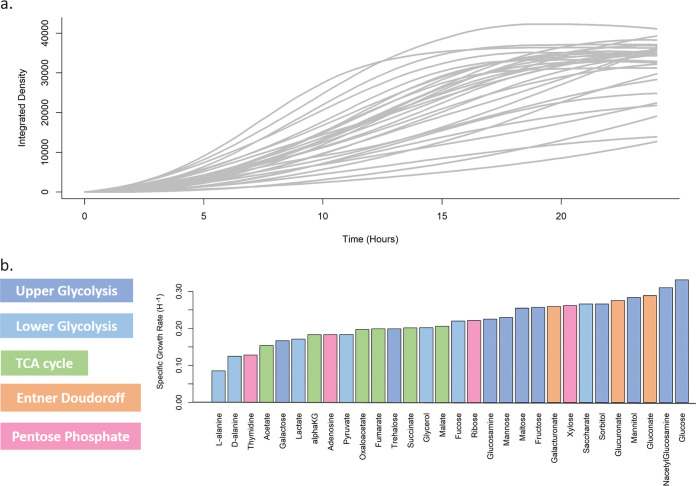
Dynamic analysis of growth in various carbon sources. (a) The growth curves of E. coli for each carbon source were calculated by taking the interquartile mean (40) of integrated density of every colony at each time point. We have called this the WT phenotype because it is generated from many strains that are unperturbed for growth by mutation. Each carbon source is unique in how they affect the growth of E. coli. (b) Average growth rate was calculated for each carbon source based on these growth curves in a by taking the maximum slope of the log-transformed graph. The rates are plotted from slowest to fastest, left to right.

It is well known that carbon source quality affects the growth rate of E. coli. We compared the growth rates of E. coli grown in different carbon sources under our screening conditions. In our screen, most conditions only showed around 100 phenotypes (Table 1), meaning most of the genes in E. coli are not directly associated with a carbon source phenotype. Based on this, we can assume that most mutants will grow the same as a wild-type (WT) strain. By using the interquartile mean (mean of the middle 50% of rank-ordered data for each condition—see Materials and Methods) for every mutant at every time point (*n* = 1,898), we were able to generate a representative WT growth curve on each carbon source (Fig. 3a). Indeed, this curve serves as an internal control and is representative of the exact conditions to which every mutant in the screen was subjected. To confirm that this assumption was valid, we investigated the growth of WT E. coli in five different carbon sources and found close correspondence with the growth of the unperturbed mutants using the interquartile mean approach (Fig. S3). Using this growth curve, we calculated the maximum growth rate of E. coli grown on each carbon source (Fig. 3b). As expected, growth on glucose as the sole carbon source shows the highest growth rate. For E. coli, catabolite repression is activated during growth on glucose to ensure it is preferentially metabolized. (9). In general, we found that E. coli grows well in glycolytic sugars and hexuronates that are degraded using the Entner-Doudoroff pathway. Oxaloacetate, fumarate, succinate, and malate are all TCA cycle intermediates which had similar growth rates. Of note, l-alanine has a lower growth rate than d-alanine in E. coli. In order for E. coli to use l-alanine as the sole carbon source, it must first be converted into d-alanine through the activity of alanine racemase. This may be a bottleneck and account for the difference in growth rate. There are two alanine racemase enzymes in E. coli, which are encoded by the genes *alr* and *dadX.* While *alr* is constitutively expressed, *dadX* is the predominant isozyme that is responsible for most of the degradation activity in the cell (41). This is reflected in our data where the *Δalr* mutant is able to grow on l-alanine as the sole carbon source, but there is no growth from the *ΔdadX* mutant on l-alanine.

### Carbon Phenotype Explorer.

**FIG 4:**
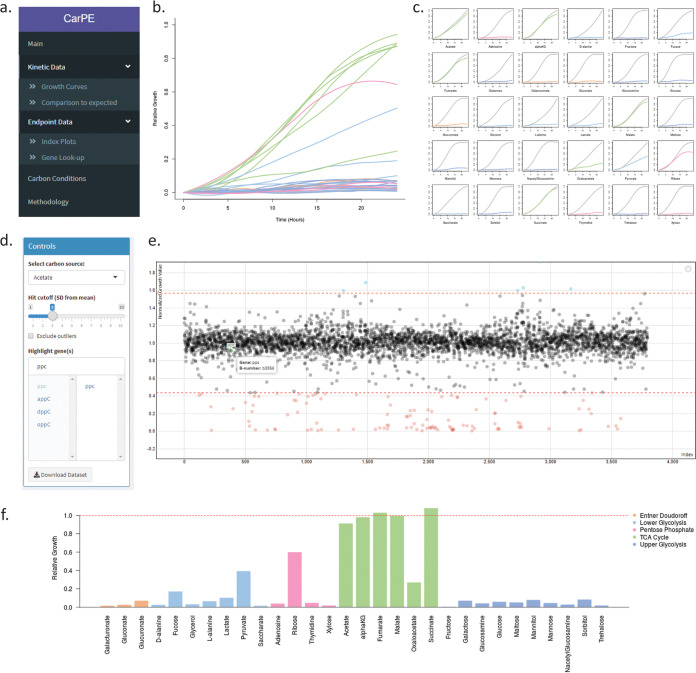
Carbon Phenotype Explorer (CarPE) Web application. Example screen shots for the CarPE Web application are shown (https://edbrownlab.shinyapps.io/CarPE/). Each of the example plots were made based on the phenotypes for the gene *ppc.* (a) Shown are the sidebar links to the different visualization tools. (b) An example plot showing the growth curves of the *Δppc* deletion mutant in each of the 30 carbon sources. Users can select which carbon source conditions to show and download the data used to make such plots. (c) Shows that the growth curves of the mutant can be compared individually with the plot of the WT control for each carbon source. (d) Here, a control panel is shown that allows users to change the parameters of the index plot (e) and highlight genes of interest. (e) An example of an index plot is shown. The index plot shows the growth of every mutant strain under a carbon condition. The genes in the index plot are in the order our collection was arrayed, showing that positional effects were normalized out. Users can hover over the plot to identify the gene name and B-number. (f) A bar plot that shows the relative growth of the selected mutant strain across all carbon source conditions.

In our experiment, kinetic measurements were obtained at 20-minute intervals, adding up to over 8 million data points collected which generated 113,880 individual growth curves for each mutant under every condition. To facilitate the accessibility of these data, we created a Web application that allows for easy visualization of the growth curves for each gene deletion mutant (https://edbrownlab.shinyapps.io/CarPE/). In this application, users can visualize any of the growth curves that were generated in this study by selecting the gene and carbon sources of interest (Fig. 4). There are two ways that we have plotted these data; one where the relative growth in each carbon source is on the same graph, and another where each mutant is compared with the expected WT curve for each carbon source. We observed that in certain carbon sources, the edge colonies grew larger than center colonies. This is a phenomenon often seen in solid medium screens where neighbor effects due to competition for nutrients may occur (27, 28). To account for these edge effects, colonies that were on the edge of the plate were instead plotted against the interquartile mean of all edge colonies in that condition.

Our data explorer also allows for visualization of the 24 h endpoint growth values, which is a function of colony size (27). Users can select a carbon condition of interest and see the relative growth of all mutants represented in an index plot. This interactive plot allows the user to zoom into different sections of the graph and visualize the normalized growth value of each gene under a specific condition. Hovering above each point shows a tool-tip of the gene name and Blattner number (B-number) of the data point. Gene names can be selected and highlighted on the plot with labels so the user can see its growth compared with that of the rest of the mutants. Based on a user-selected cutoff value, a table of gene names and their corresponding Gene Ontology GO term annotations appear on a table underneath so users can see which gene mutants showed significantly low or high growth in each carbon source. Where valid, these genes are linked to the EcoCyc webpage which contains curated information about the gene (23). For comparing between conditions, we have included a function where the user can type in a gene and look at the normalized growth under each condition plotted as a bar graph. Since the mutants in the Keio collection contain a kanamycin cassette, this may cause polar effects on downstream genes (11). Using CarPE, the user can look up genes downstream of their gene of interest and determine whether they have a similar carbon profile. Considering these functionalities, this Web application allows other users to easily navigate and analyze our data set.

### Comparisons to the EcoCyc metabolic model.

We wanted to evaluate whether the phenotypes that we observed matched the current knowledge of E. coli central carbon metabolism. We compared our experimental data with the predicted growth phenotypes using the EcoCyc metabolic model (23, 42). Using flux balance analysis, the medium composition is considered and the model can predict growth in each gene knockout. To simulate a gene knockout in the model, reactions associated with that gene are compiled using the EcoCyc database. The model considers the relationships between a gene, its protein product, any complex(es) formed by the product, and the reaction(s) catalyzed by the product and/or complex. The model then simulates the experiment by attempting to use the remaining reactions to generate the full set of biomass metabolites required for growth. If any of these metabolites are no longer produced, or if the predicted growth rate is below our cutoff, the simulation will produce a no-growth result. There were 1,402 genes that overlapped between our data set and the model (see Table S4 in the supplemental material). On average, the model predicted the same phenotype as our experiments with 95% accuracy (Table 2). By comparing our data with the model, we can identify genes that show a growth discrepancy that should be focused on for further study. Genes that had a predicted growth rate above our cutoff of 1 × 10^−3^/h were predicted to be dispensable in the model. If that same gene deletion mutant failed to show growth experimentally when deleted, it was considered a false-positive prediction. Conversely, a false-negative prediction means the model predicted no growth from the mutant but our experimental data showed growth. We identified 33 genes that were found to have either a false-positive or -negative prediction across all carbon sources (see Fig. S4 in the supplemental material). Of these genes, seven were determined to be essential for growth in previous studies (12). An update to the Keio collection showed that these mutants contained a gene duplication which explains why these mutants grew in our experiment despite being essential for growth in rich media (43). We repicked the remaining 26 genes and grew them in liquid media to determine whether these phenotypes were artifacts from high-throughput screening. Out of these genes, only nine that showed growth in solid media did not in liquid media (Fig. S4). These genes were all involved in vitamin biosynthesis pathways. It is interesting to note that apart from the Δ*pdxH* mutant, these mutants exhibited modest growth until they were passaged in minimal media.

**TABLE 2:** Experimental phenotypes compared with the EcoCyc metabolic model predictions^*a*^

Experimental result	No. of phenotypes matching model prediction	
	Growth	No growth
Growth	38,362	1,167
No growth	645	1,886

a The model predicted the same phenotype as experiments with 95.7% accuracy. Also, breakdown of the accuracy in each carbon source can be found in Table S6.

10.1128/mBio.02259-20.4 FIG S4 Flow chart comparing the 33 genes we have identified in this study that do not fit in the model under any condition. Seven of the mutants contain a gene duplication which allows the mutant to grow. Nine of the genes were involved in vitamin biosynthesis and did not grow in liquid media. The remaining 17 genes were compared to 2 other gene essentiality data sets previously published (11, 14, 43). Download FIG S4, TIF file, 0.1 MB. Copyright © 2020 Tong et al. 2020 Tong et al. This content is distributed under the terms of the Creative Commons Attribution 4.0 International license.

10.1128/mBio.02259-20.8 TABLE S4 A total of 1,402 genes in the EcoCyc model compared with experimental results. Download Table S4, XLSX file, 0.1 MB. Copyright © 2020 Tong et al. 2020 Tong et al. This content is distributed under the terms of the Creative Commons Attribution 4.0 International license.

We also identified 198 genes that have a false prediction in at least one carbon source (see Table S5 in the supplemental material). One of the discrepancies identified in this study is the inability of the model to correctly predict the growth patterns of a *Δppc* mutant. The enzyme encoded by *ppc*, phosphoenolpyruvate (PEP) carboxylase, catalyzes an anapleurotic reaction which converts PEP into oxaloacetate and replenishes it in the TCA cycle (44). The model predicts that the cell is able to grow without this enzyme by activating the glyoxylate shunt and generating oxaloacetate through that pathway instead (42). Our data show that the *Δppc* mutant can only grow when the sole carbon source is one of acetate, α-ketoglutarate, fumarate, malate, succinate, or ribose. This finding means that during growth in most carbon sources, this enzyme is vital for regulating the levels of PEP and oxaloacetate in the cell, but with different TCA cycle intermediates, notably not oxaloacetate, this is no longer necessary. Thus, by screening a large number of carbon sources, we have characterized enigmatic patterns of regulation which may not be obvious in a smaller sample size of carbon sources. Comparing such data to a metabolic model can generate new insights for genes that have already been well studied.

10.1128/mBio.02259-20.9 TABLE S5 A total of 198 genes with at least 1 false prediction in any carbon source. Download Table S5, XLSX file, 0.03 MB. Copyright © 2020 Tong et al. 2020 Tong et al. This content is distributed under the terms of the Creative Commons Attribution 4.0 International license.

10.1128/mBio.02259-20.10 TABLE S6 Accuracy of the model in predictions for each carbon source. Download Table S6, XLSX file, 0.01 MB. Copyright © 2020 Tong et al. 2020 Tong et al. This content is distributed under the terms of the Creative Commons Attribution 4.0 International license.

### YdhC is a putative transporter involved in adenosine export.

**FIG 5:**
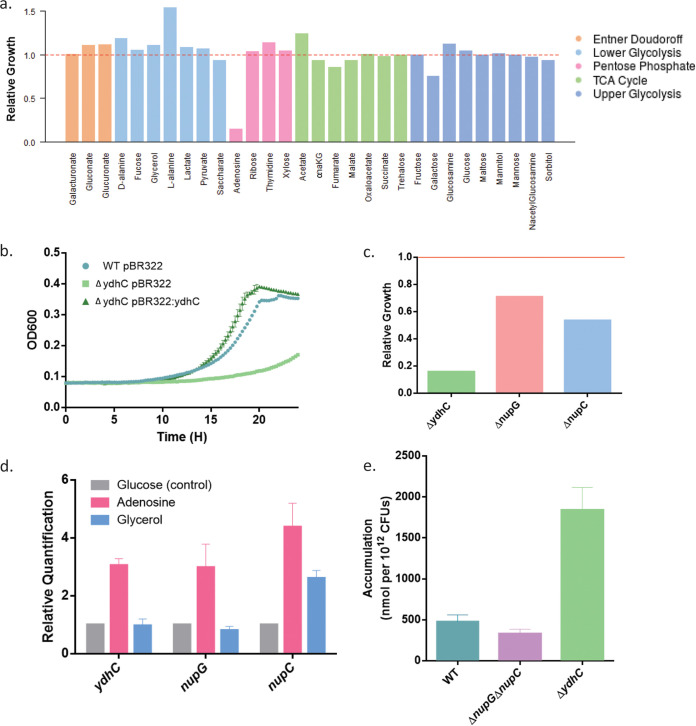
Hypothesis generation and analysis of the function of gene *ydhC*. (a) Growth profile of the *ΔydhC* mutant in all carbon sources, highlighting the specific growth defect when adenosine is the carbon source. (b) Growth curves in MOPS minimal media containing adenosine as a carbon source. The growth defect of a *ΔydhC* mutant disappears when complemented with a plasmid containing the gene. Shown is a representative example of many replicates. (c) Comparison of the final growth amplitudes of three deletion mutants, namely, Δ*nupC*, Δ*nupG*, and Δ*ydhC*, that have been implicated in adenosine transport. (d) Reverse transcription-quantitative PCR (qRT-PCR) data showing the fold change in response (threshold cycle [ΔΔ*CT*] values) for each gene (*nupC*, *nupG* and *ydhC*), relative to that in glucose, when cultured in glycerol and adenosine, respectively. The reference gene was E. coli 16S rRNA. (e) Accumulation assays measuring the amount of adenosine in cells that were treated with adenosine for 10 minutes. Cells were lysed and adenosine levels were measured using LC-MS as described in the Materials and Methods.

Despite the many efforts to fully annotate every gene in E. coli, 35% of the genes still lack experimentally defined functions (45). We can use this data set to generate hypotheses for the function of some of these genes. We identified 51 genes that were poorly annotated and exhibited a growth defect on at least 1 carbon source (Table S2B). One of these genes was *ydhC*, which codes for a putative transporter of the major facilitator superfamily (MFS) (46). We noted slow growth for the *ΔydhC* mutant specifically when grown on adenosine as the sole carbon source (Fig. 5a). This gene is found downstream of the gene *purR*, a regulator of purine biosynthesis. Interestingly, Sastry et al. (47) recently applied unsupervised learning to transcriptome sequencing (RNA-seq) data sets and identified *ydhC* as a potential member of the *purR* regulon. We elected to follow up on *ydhC* to identify its involvement in adenosine metabolism.

To ensure that the finding of slow growth for the *ydhC* mutant was not an error due to the high-throughput nature of our screen, we remade the deletion mutant using homologous recombination with an apramycin resistant marker. We then checked to see if we could complement back this phenotype by cloning the gene onto a plasmid. Since there is no annotated promoter for *ydhC*, we cloned this gene, complete with a section 200 bp upstream, into a pBR322 plasmid. Compared with the empty plasmid vector, we found that the growth of the Δ*ydhC* mutant returned to wild-type levels in the presence of this plasmid (Fig. 5b).

Since YdhC is annotated as a transporter in the major facilitator superfamily (MFS), we postulated that YdhC may be involved in adenosine transport (46). Interestingly, two genes, namely, *nupG* and *nupC*, have already been shown to code for nucleoside transporters. In our study, we found that *ΔydhC* has the strongest growth defect when using adenosine as a sole carbon source (Fig. 5c). Consistent with both *nupG* and *nupC* coding for purine nucleoside transporters, a single deletion in either of these genes could be compensated by the presence of the other. The fact that Δ*ydhC* showed such a strong growth defect by itself implied it was not a redundant function of the same adenosine transport system. Notably, a Δ*nupG*Δ*nupC* double mutant could not grow in media with adenosine as a sole carbon source (48). We reasoned that if *ydhC* was important in adenosine metabolism, it should be upregulated in the presence of adenosine. To test this hypothesis, we performed a reverse transcriptase quantitative PCR (RT-qPCR) experiment to see if the *ydhC* transcript levels increased after treatment with adenosine. As a control, we also tested *nupG* and *nupC* transcript levels, as these genes are expected to be upregulated in the presence of adenosine. Of course, when E. coli grows on glucose, there is catabolite repression and transport of other carbon sources is downregulated (9). To ensure our increased expression was not due to the lack of catabolite repression and was indeed specific to adenosine, we also tested glycerol as a control carbon source. We grew WT E. coli cells for 2 hours to mid-exponential phase, washed them, and then treated them for 1 hour in glucose, glycerol, or adenosine. We isolated the RNA from these cells and then measured the expression levels of the genes *ydhC*, *nupG*, and *nupC*. We found that they all showed increased levels in adenosine compared with those of glucose and glycerol controls (Fig. 5d).

Since YdhC has previously been implicated in arabinose efflux (49), we performed an accumulation assay to determine whether adenosine would be one of its efflux substrates (50). If YdhC is an efflux transporter, deletion of this protein would result in the increase of intracellular concentrations of adenosine since the cell would no longer excrete it. We grew *ΔydhC* and Δ*nupG*Δ*nupC* mutants to mid-log-phase growth and treated them with adenosine for 10 min. The cells were washed and the cell lysates were then collected. We then used liquid chromatography-tandem mass spectrometry (LC-MS/MS) to quantify the amount of adenosine that was present in the cells. The Δ*ydhC* mutant accumulated more adenosine than the wild-type, implicating it in adenosine efflux (Fig. 5e). This finding is a proof-of-principle example of the utility of our data set for generating hypotheses for the functions of poorly annotated genes.

## DISCUSSION

Our primary goal was to generate a biologically meaningful data set to further our understanding of E. coli carbon utilization patterns and central metabolism. Here, we have characterized the growth of each nonessential gene deletion mutant in E. coli grown on 30 different carbon sources. The carbon sources chosen probe all of the metabolic pathways within central metabolism and allow insights into aspects of E. coli physiology that are linked to carbon metabolism. The data collected in this study largely correlate with the EcoCyc metabolic model, which reflects our current knowledge in central metabolism. Nevertheless, we have noted several paradoxes and provide a list of genes with discrepant phenotypes, highlighting gaps in our understanding and providing a starting point for advancing current metabolic models. We developed a Web-based application that allows for easy visualization of the data for researchers interested in querying phenotypes and investigating genes of interest. We hope that this tool will facilitate the generation of hypotheses for the functions of enigmatic genes and help efforts in functional genomics related to carbon source utilization. In proof-of-principle efforts, we showed here how these phenotypes helped us to describe YdhC as an adenosine efflux transporter in the major facilitator superfamily.

Although E. coli can grow on a large number of carbon sources, the quality of the carbon sources will impact the growth rate (10). The preferential usage of glucose and the effect of carbon catabolite repression is well-documented in previous studies (9). The latter is important for E. coli to grow with the preferred sugar when faced with multiple choices in a natural environment. Apart from glucose, however, the preferred carbon sources of E. coli are not well studied. A previous study looked at the activity of different sugar promoters in mixtures of carbon sources to determine a hierarchy of sugar utilization. The researchers noted that in a mix of two carbon sources, the promoter for the carbon source that supports a higher growth rate has higher activity, while the less dominant sugar shows reduced activity (51). In this study, we described a hierarchy of carbon source utilization based on growth rate. We saw that this was not dependent on the pathway of central metabolism to which the carbon source was metabolized. Ribose, xylose, adenosine, and thymidine are all metabolized into intermediates in the pentose phosphate pathway. However, we show that E. coli has different maximum growth rates on these carbon sources. It would be interesting to combine different carbon sources systematically to discern how E. coli chooses its preferred carbon source given a mixture and whether this correlates with the growth rate in the sole carbon source. This would have implications in host infection environments where E. coli and other pathogens may encounter many different potential carbon sources.

Compared with other hexose sugars, we noticed that growth on galactose is slow. This observation was noted in a previous study characterizing the MG1655 strain of E. coli (52). Soupene et al. (52) adapted a culture of E. coli to grow quickly in galactose and found that the *lac* operon was induced in that culture. Since LacY, a lactose transporter, can also transport galactose, these mutations likely caused an increase of galactose uptake. They also identified an induction in the *nag* operon, a set of genes involved in *N*-acetylglucosamine metabolism, suggesting the cross-regulation between galactose and *N*-acetylglucosamine catabolic pathways. Qaidi et al. (53) further studied this phenotype and showed that NagC is involved in repressing *galP*, a gene that codes for galactose permease (53). In this study, we identified similar deletion mutants that showed improved growth, including *galR*, *galS*, and *nagC*. All three of these gene products repress galactose transport, which means that deleting these genes increases galactose uptake into the cell. Based on these findings, the growth rate using galactose appears to be limited by its transport into the cell. The presence of galactose has been known to induce stress in E. coli *ΔgalE* (galactose epimerase) mutants (54). While our study only looked at galactose as the sole carbon source, other researchers have noted that the addition of galactose, even when another carbon source was present, can cause growth inhibition in these mutants (55). In Bacillus subtilis, accumulation of the toxic intermediate UDP-galactose imparts a growth defect on the cell (56). In E. coli, UDP-galactose is used in lipopolysaccharide (LPS) biosynthesis, both in the core and O-antigen. E. coli K-12 strains do not synthesize O-antigen since there are mutations in the operon. Given that accumulation of UDP-galactose is toxic to the cell, E. coli strains that do not synthesize O-antigen may limit galactose transport into the cell since there are fewer pathways available for metabolizing UDP-galactose. Indeed, we noticed that clinical strains of E. coli that synthesize O-antigen have a higher growth rate on galactose than our K-12 strains (data not shown).

Our experimental data matched the predicted phenotypes from the EcoCyc metabolic model with 95.7% accuracy (Table 1). In total, we identified 198 genes with a false prediction in at least one carbon source. Resolving these inconsistencies is an opportunity to amend models that may be missing isozymes or contain incorrect annotations. Guzmán et al. (57) recently followed up on a list of genes that showed a false-positive prediction in a previous study. They showed that some of these mutants started growing when they increased the length of the experiment past the typical time point of 24 h. Furthermore, they highlight how studying inconsistencies between the model predictions and experiments can reveal important physiological features of E. coli, such as its genetic and metabolic flexibility in overcoming its gene deletions (57).

We initially identified a set of 33 genes which do not match computational predictions in any of the carbon sources tested. Out of these paradoxes, seven were identified in gene deletion strains of the Keio collection that have since been shown to be flawed because of gene duplication events (43). When we rescreened the remaining 26 mutants, 9 of them did not grow in liquid cultures of MOPS minimal media with glucose or glycerol as a carbon source. These nine mutants all corresponded to genes involved in vitamin biosynthesis. Vitamin requirements for E. coli growth can be extremely low; for example, an E. coli *ΔbioA* mutant requires only 0.2 to 0.4 ng/ml to restore growth (58). Because trace amounts of vitamins can supplement the growth of these auxotrophs, we suggest that it can lead to variable growth findings. One of the limitations of using solid media for our screen is the necessity of growing the mutant colonies in close proximity. Because of this proximity, true auxotrophs with low nutrient requirements may be able to grow on the trace amounts released by neighboring colonies. Nevertheless, this problem appears to be unique to genes involved in vitamin biosynthesis pathways since amino acid auxotrophs largely showed no growth in our screen. Of the 17 remaining paradoxical genes, we compared our findings for these to 2 other data sets, in which 1 uses glucose (11) as the sole carbon source and another uses glycerol (14). Our experimental findings for these 17 genes matched the growth patterns found in at least 1 of these other data sets (Fig. S4). Some 11 of these deletion mutants showed inconsistent growth across data sets, and as a result, it is difficult to determine their phenotypes in minimal media. Six of these genes (i.e., *pabC*, *zupT*, *kdsC*, *fpr*, *ubiC*, *and pyrI*) reproducibly showed growth in all three data sets. These genes were incorrectly predicted by the EcoCyc metabolic model to be essential on all carbon sources, and resolving these inconsistencies may help improve the model. One of the genes, *zupT*, encodes a heavy metal divalent cation transporter and was predicted to be essential in all carbon sources. It was predicted to be essential because it was set to be the only reaction which allows the organism to obtain metal cations. However, this is surely not the case, as E. coli can grow in the absence of this transporter. In all, a careful comparison of metabolic modeling with a large number of experimental phenotypes is a promising starting point for generating hypotheses about E. coli physiology and identifying missing reactions in the current metabolic models.

While metabolic models are good at predicting which genes are required, they sometimes fail to account for differences in regulation. In our experimental results, for example, we found that the *ΔsrlA* and *ΔsrlE* mutants were unable to grow in sorbitol as a sole carbon source. These genes code for subunits of the sorbitol-specific phosphotransferase system (PTS) enzyme. The model predicts growth for these genes due to the presence of the *gatABC* genes which encode the galactitol-specific PTS enzyme. This enzyme is able to transport sorbitol with low affinity; however, in some E. coli strains, this gene is induced by galactitol and is likely not expressed at a high enough level when grown in sorbitol as a sole carbon source (59). Since the model is unable to account for regulation, it predicts that sorbitol can enter the cell using a secondary transporter which may not be expressed under the given condition. We noticed that the *Δppc* mutant shows a growth defect in most carbon sources except for most of the TCA cycle intermediates, namely, succinate, fumarate, malate, acetate, and α-ketoglutarate. In contrast, the flux balance model predicts growth through activation of the glyoxylate shunt to generate oxaloacetate under all conditions (60). Ppc catalyzes an anapleurotic reaction which generates oxaloacetate from phosphoenolpyruvate. During growth on most carbon sources, this reaction is required to replenish oxaloacetate, as TCA cycle intermediates get used for biosynthesis of amino acids. However, when E. coli is grown on TCA cycle intermediates as a sole carbon source, there is no longer a need to generate more oxaloacetate and Ppc is not required for robust growth. Since there are other pathways to generate oxaloacetate, it is not readily obvious when this regulatory enzyme is required for growth. By testing a *Δppc* mutant for growth across carbon conditions, we have identified specific conditions where this enzyme is important for growth and revealed a new aspect of E. coli metabolism.

Metabolite efflux is well-described in the literature (49, 61–64). In 1980, Huber et al. (65) found that a large proportion of β-galactosidase products, galactose, glucose, and allolactose, were found in the culture media when lactose was added. Cells were found to be intact, and no β-galactosidase was detected in the media. Furthermore, this finding was only observed when the lactose permease was present and lactose can be imported into the cell. This implies that lactose was imported into the cell and broken down by β-galactosidase and the products were excreted into the media as they were generated during growth. Subsequently, Liu et al. (64) described a family of β-galactoside sugar efflux pumps (*setABC*) in E. coli which explained this observation. For other sugars, studies have implicated YdeA and YdhC in arabinose efflux (49, 66). Interestingly, both arabinose and adenosine are metabolized into ribulose-5-phosphate in the pentose phosphate pathway. Previous studies have shown that the accumulation of ribulose-5-phosphate results in a growth defect (67). This may explain why E. coli has a need for controlling intracellular levels of these carbon sources. In E. coli, gene *nepI* has recently been characterized to efflux inosine, setting a precedent for nucleoside export (62). However, unlike *ydhC*, a *ΔnepI* mutant did not appear to have a growth defect when grown in inosine or adenosine.

Current antibiotics target a small subset of cellular processes that are deemed to be essential; however, essentiality is contextual and dependent on the growth environment (13). There has been growing interest in targeting genes important under nutrient-restricted conditions that may also be required for infection *in vivo* (2, 6). Additionally, there has been interest in targeting bacterial carbon utilization pathways, as there is recognition that various carbon sources may have different levels of importance at different sites of infection (2–4, 68, 69). In our study, we identified 342 genes that were conditionally essential dependent on the carbon source available and 74 genes that were essential in MOPS minimal media regardless of carbon source. Although knowledge of the nutrient composition available to pathogens at various sites of infection is an emerging field of study (6, 58, 70, 71), the data generated here may have value to help us understand the set of unique targets that are practicable in the context of antibacterial drug discovery. This information could generate new hypotheses for drug target validation studies and could catalyze the creation of unique screening platforms for identifying compounds that are uniquely antibacterial at specific sites of infections (72–74). Indeed, a carbon source and site-specific approach to antibacterial therapy might have the benefit of sparing commensal bacteria that use a different carbon source. Additionally, by understanding the genes required for growth that is carbon source-specific, we might avoid prioritizing targets that are not relevant *in vivo.* For example, Mycobacterium tuberculosis grown under standard laboratory conditions uses glycerol as a carbon source. A screen for antitubercular compounds was performed in this medium and identified compounds targeting glycerol metabolism which showed potent *in vitro* activity (74). However, since glycerol was not a relevant carbon source *in vivo* for M. tuberculosis, the compounds lacked any *in vivo* efficacy (74). Therefore, understanding bacterial carbon source utilization has foundational value in the hunt for new antibiotics. Similar studies in various commensals and pathogens will likewise provide a useful data set with the capacity to identify targets that are pathogen specific.

In conclusion, we have generated an easily accessible set of data that will be broadly useful in microbiological research. This study provides a starting point for future investigations aimed at further understanding bacterial central metabolism.

## MATERIALS AND METHODS

### Chemicals.

Chemicals used in this study were purchased from Sigma-Aldrich unless otherwise noted. A full list of carbon sources and the concentrations can be found on the carbon conditions tab in the Carbon Phenotype Explorer (https://edbrownlab.shinyapps.io/CarPE/). Concentrations were picked so that all carbon sources resulted in the same amount of carbon added. MOPS minimal media (Teknova) was used for all work in minimal media. Antibiotics were added to the medium as required with final concentrations as follows: 50 μg/ml, kanamycin; 100 μg/ml, ampicillin; 25 μg/ml, chloramphenicol; and 100 μg/ml, apramycin.

### Bacterial strains and growth conditions.

In this study, we screened the Keio collection of E. coli K-12 nonessential gene deletions (11) and 100 small RNA (sRNA) and small protein deletions (18) in 30 different carbon sources. The sRNA and small protein deletion library were generated in E. coli strain MG1655 (F^−^ LAM^−^ *rph-1*), while the Keio collection was in E. coli strain BW25113 [F^−^ Δ(*araD*-*araB*)*567 lacZ4787*Δ::*rrnB-3* LAM^−^ *rph-1* Δ(*rhaD*-*rhaB*)*568 hsdR514*].

Strains that did not show consistent growth across repeated screens were removed from this study. In our final data set, we have gathered information on 3,796 strains of E. coli.

For routine experiments, the E. coli BW25113 strain was streaked onto LB media with appropriate antibiotic selection from a frozen glycerol stock and grown overnight in a stationary incubator at 37°C. A single colony was picked to inoculate an overnight culture which was grown shaking at 250 rpm. This culture was used to subculture into a new tube to mid-log-phase growth. For experiments in minimal media, this subculture was washed using MOPS minimal media containing no carbon source and diluted 1:100 in final conditions. Mutants were made by homologous recombination, as described by Datsenko et al. (17). Gene deletions were made by replacing the gene with an apramycin or chloramphenicol-resistant cassette. The plasmids pSET152 and pKD3 were used as the templates for the apramycin and chloramphenicol resistance genes, respectively. Deletions were moved into different mutant strains to generate double deletions by using P1 phage transduction.

### Biolog phenotype microarrays.

Colonies were picked and resuspended in M9 minimal media containing no carbon source. Phenotypic microarray assay plates (Biolog Inc., Hayward, CA) were inoculated with mid-log-phase culture of E. coli washed with MOPS minimal media with tetrazolium dye mix A (Biolog Inc.). Plates were then incubated at 37°C in a stationary incubator for 24 h and 48 h. The optical density at 600 nm (OD_600_) values were read on the Tecan plate reader at both time points. Plates were also scanned using the Epson V750 scanner to visualize the plate by eye.

### Screening conditions.

The E. coli Keio collection was pinned onto LB agar medium containing 50 μg/ml kanamycin from frozen glycerol stocks in 96-colony-density, meaning they were arrayed so that 96 colonies were on the agar plate. Colonies were grown overnight at 37°C, upscaled to 384-colony-density using a Rotor HDA (Singer Instruments), and grown overnight at 37°C. From 384-colony-density, the colonies were once again upscaled to 1,536-colony-density and grown overnight. Plates were duplicated at 1,536-colony-density in LB agar and grown overnight, and the resulting plates were stored at 4°C for no longer than 4 weeks.

Keio plates were pinned from LB agar plates onto solid MOPS minimal media containing a carbon source in 1,536-colony-density. Plates were incubated for 24 h to deplete any nutrients that may have been carried over from the LB agar. These plates were used to inoculate new solid minimal media assay plates containing the same carbon source. The assay plates were tested in two technical replicates, both at 1,536-colony-density. Assay plates were incubated for 24 h at 37°C and scanned once every 20 min using the Epson Perfection V750 scanner. The workflow is summarized in Fig. S2.

### Data analysis.

Images were analyzed using ImageJ and quantified into an integrated density value using the method described by French et al. (27). The integrated density value, a value that tracks with cell number linearly, was used as a proxy for growth at each time point. The resulting data were compiled using a program written in R. For the endpoint values, the first time point was subtracted from the last time point to remove any background noise caused by a large initial inoculum. After this step, the data were normalized as described previously by French et al. to remove edge effects (27). The raw integrated density values of each colony were divided by the interquartile mean of the row and then by the column in which it belonged. Since corner colonies grew differently from the edges, we normalized them by dividing the raw data of each one to the interquartile mean of all the corner colonies under the condition.

To ensure acceptable data quality, the glucose condition was repeated every week to check that the growth of certain strains did not change over time and through subsequent passaging of the library. There were a few strains that developed mutations which allowed them to grow in minimal media after 4 weeks of passaging. As these genes did not replicate under the same conditions, they were removed from the final data set. In addition, the genes that showed no growth in LB (the media that the collection was made and grown in) were also removed.

To determine which genes were essential under each condition, we used a cutoff that was 3 standard deviations below the mean of the data set. Mutants that grew to a normalized value below this were considered to show a low growth phenotype. Venn diagrams were drawn using a Web tool developed by the bioinformatics and evolutionary genomics group at Ghent University (http://bioinformatics.psb.ugent.be/webtools/Venn/). The COG annotation index was used to group genes according to functions (https://www.ncbi.nlm.nih.gov/COG/). We manually curated genes based on current COG annotations and existing literature published on the genes. Genes were assigned to only one COG category.

Growth curves were corrected using a local regression method (loess). Maximum growth rates were calculated by finding the maximum slope of the line of best fit between five points during log-phase growth of the log-transformed growth curves. The heatmap and clustering were constructed using the heatmap.2 function from the “gplots” package in R.

### Carbon Phenotype Explorer.

The interactive Web application developed for easy visualization of our data was built using the R “shiny” and “shinydashboard” packages. The interactive scatterplots were made using the “scatterD3” package. To analyze our kinetic data, we subtracted the first time point of each curve to minimize background noise from a high inoculum. We then took the average of our two replicates at each time point. Given that most of our data normalize to the same growth value of one, we can make the assumption that most gene mutations do not affect the growth of E. coli. (40, 75). If we take the interquartile mean of each time point, we can generate an expected curve for each carbon source. Since the low- and high-growing mutants are removed before taking the mean, this expected curve represents how wild-type bacteria would grow under a given condition. To confirm this idea, we also analyzed a plate of WT colonies arrayed on 5 different carbon sources and compared their growth to that of the unperturbed mutants using the interquartile mean approach. Since different carbon conditions grew to different final colony sizes, we normalized the data by dividing each value by the maximum value of the expected curve. This normalizes the data from each time point to a relative growth value based on what we expect a typical colony to grow to under the conditions. This allows different conditions to be compared since curves are now normalized to a similar final amplitude.

### Flux Balance Analysis.

We used the flux balance analysis (FBA)-based EcoCyc metabolic model for E. coli strain BW25113 and the data from EcoCyc version 22.0 to obtain growth/no-growth predictions on each carbon source (23, 76). The cutoff for growth for the predictions was set to anything greater than 1 × 10^−3^/h. The carbon source uptake rate was set to 10 mmol/g of dry weight (gDW)/h under aerobic conditions. The nutrients provided were set to be the same as the ingredients of MOPS minimal media. There were 1,427 genes in the model, and they were tested against the 30 carbon sources used in this study.

### Liquid growth kinetics.

Phenotypes were reconfirmed by testing their growth in liquid media in a 96-well plate. Overnight incubations of the strain of interest were subcultured in LB and grown until an OD_600_ of 0.4 was reached. Cells were subsequently washed in MOPS minimal media with no carbon source and diluted 1:100 into the carbon source of interest. OD_600_ measurements were taken every 15 min for up to 48 h using a Sunrise absorbance microplate reader (Tecan Life Sciences).

### Construction of complementation plasmid.

The construction of the pBR322 plasmid containing *ydhC* was performed using standard DNA manipulation techniques. DNA encoding the gene was obtained by PCR amplification from E. coli genomic DNA (forward, 5′ ATGACCGGATCCGAACGCGATGCAGCTCCTGTG 3′; reverse, 5′ATGACCGAATTCGTCTATAATCCGACGTAGAAC 3′). This plasmid was constructed by inserting the *ydhC* gene along with 200 bp upstream of the gene into the EcoRI and BamHI sites on the plasmid. Both the PCR products and the pBR322 plasmid were digested using EcoRI and BamHI enzymes, gel purified using the gel extraction kit (Qiagen, Hilden, Germany), and ligated using T4 ligase.

### RNA isolation, cDNA preparation, and quantitative RT-PCR.

Bacterial cultures were grown to an OD of 0.4 in LB media. Cells were washed three times in MOPS minimal media containing no carbon source. The washed culture was split into three media containing different carbon sources, namely, glucose, glycerol, and adenosine. They were incubated at 37°C for 1 hour before the cells were harvested and added to ice-cold 5% acid phenol in ethanol solution. Cells were centrifuged for 10 minutes at 4°C, and the resulting cell pellets were used for RNA extraction. RNA was extracted using the RNAzol reverse transcription (RT) RNA isolation reagent. (77) cDNA was prepared using the high-capacity cDNA reverse transcription kit (Applied Biosystems, Foster City, CA, USA).

Quantitative reverse transcription-PCRs (RT-PCRs) were run using a CFX96 real-time system (Bio-Rad). Reactions (20 μl) contained SYBR green quantitative PCR (qPCR) master mix (Dongsheng Biotech, Guangzhou, Guangdong, China), primers at a final concentration of 400 nM, and 5 ng of cDNA. 16S rRNA (rrsA) was used as a reference gene. The following primers were used for qPCR (5′ to 3′): ydhC-F, GCAGAAATGGCAAGGCAAGC; ydhC-R, GGCAATCGCCATCACACAGA; nupG-F, GCCAGTGCACAAGGGATGTT; nupG-R, GAACCACGGAGTAACCAGCG; nupC-F, AGCATCTCCTTCCAGGGCAT; nupC-R, AGATGATGCCTTCAGCACGC; 16S-F, GAAGACTGACGCTCAGGTGC; and 16S-R, GGGCCCCCGTCAATTCATTT. Cycling conditions were as follows: 95°C for 2 min, and 40 cycles of 95°C for 15 s, and 62°C for 40 s.

### Accumulation assay.

The method for quantifying l-adenosine accumulation was adapted from Richter et al. (50) with some modifications. Following equilibration of the samples at 37°C with shaking for 5 minutes, cells were treated with 12 mM l-adenosine and incubated for another 10 minutes at 37°C with shaking. A total of 800 μl of the treated cultures were layered on 700 μl of silicone oil (9 parts AR200: 1 part Sigma high temperature; cooled at –80°C). Cells were pelleted and lysed, and the supernatant was collected as described by Richter et al. (50). Supernatants were lyophilized overnight with the Virtis BenchTop Pro lyophilizer (SP Scientific), resuspended in methanol, and analyzed by LC-MS. The accumulation assay was performed in biological triplicates, and each sample was injected twice into the LC-MS.

Samples were analyzed with the LTQ Orbitrap XL MS system (Thermo Scientific) with a 1290 infinity series high-performance liquid chromatography (HPLC) system (Agilent Technologies) including a degasser, an autosampler, and a binary pump. The LC separation was performed on a HILIC-Z column (2.1 by 150 mm, 2.7 μm) (Agilent Technologies) with mobile phase A (10 mM ammonium acetate, pH 9.8) and mobile phase B (98% acetonitrile and 2% mobile phase A). The flow rate was 0.2 ml min^−1^. The gradient was as follows: 0 to 2 minutes, hold 15% mobile phase A; 2 to 17 minutes, linear gradient to 80% mobile phase A; 17.1 minutes, 15% mobile phase A and hold until 25.5 minutes. The injection volume was 10 μl. Mass spectra were acquired with positive electrospray ionization at the ion spray voltage of 3,900 V. The source temperature was 250°C. The sheath gas, auxiliary gas, and sweep gas were 40, 0, and 5 arbitrary units, respectively. Single-ion monitoring of 268.1 *m/z* was used to quantify the metabolite.
