# Erratum for Tong et al., “Gene Dispensability in Escherichia coli Grown in Thirty Different Carbon Environments”

**DOI:** 10.1128/mBio.02865-20

**Published:** 2020-11-17

**Authors:** Madeline Tong, Shawn French, Sara S. El Zahed, Wai kit Ong, Peter D. Karp, Eric D. Brown

**Affiliations:** a Michael G. DeGroote Institute for Infectious Disease Research, Department of Biochemistry and Biomedical Sciences, McMaster University, Hamilton, Ontario, Canada; b Bioinformatics Research Group, SRI International, Menlo Park, California, USA

## ERRATUM

Volume 11, no. 5, e02259-20, 2020, https://doi.org/10.1128/mBio.02259-20. On PDF page 1, in the abstract, in the sentence “From our comparisons to the model, we found that, unexpectedly, phosphoenolpyruvate carboxylase (*ppc*) was required for robust growth in most carbon sources other than most trichloroacetic acid (TCA) cycle intermediates,” “trichloroacetic acid” should be “tricarboxylic acid.” This change does not change the conclusions of the paper.

