# Supplementary figures and images for: Gene Dispensability in Escherichia coli Grown in Thirty Different Carbon Environments
Source: mBio. 2020 Sep 29;11(5):e02259-20. doi: 10.1128/mBio.02259-20 (PMC7527729; doi:10.1128/mBio.02259-20)

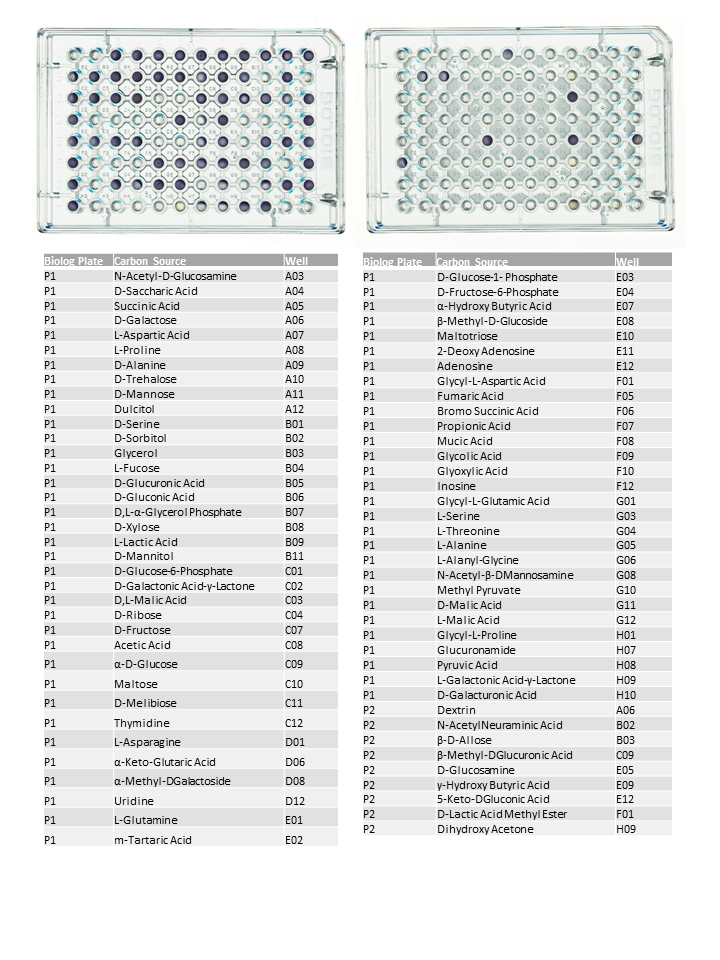

Supplement: FIG S1 [file mBio.02259-20-sf001.tif]

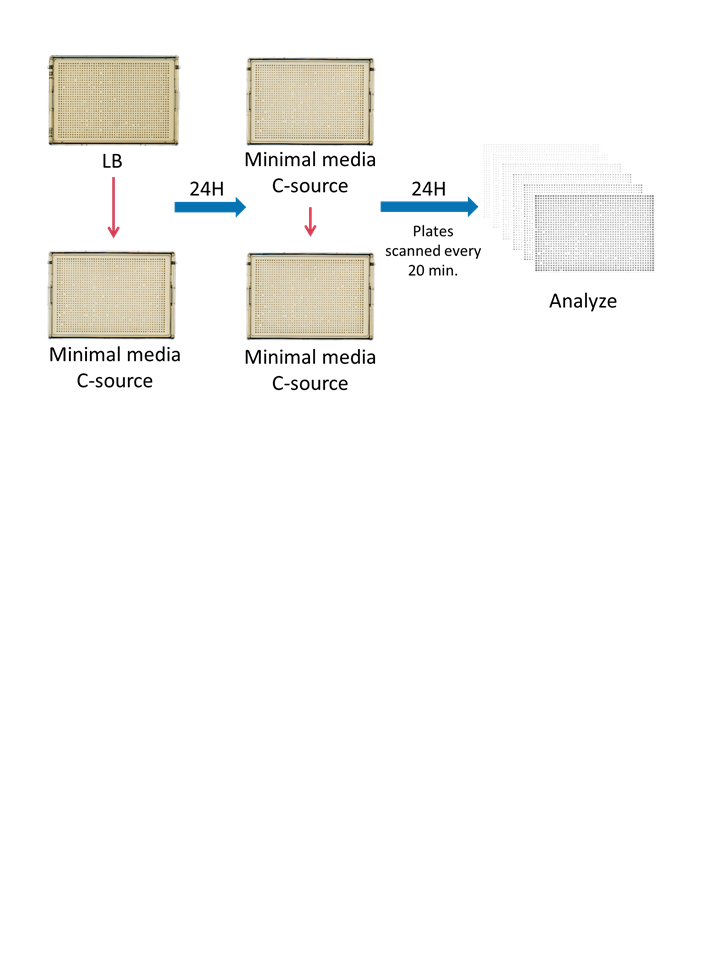

Supplement: FIG S2 [file mBio.02259-20-sf002.tif]

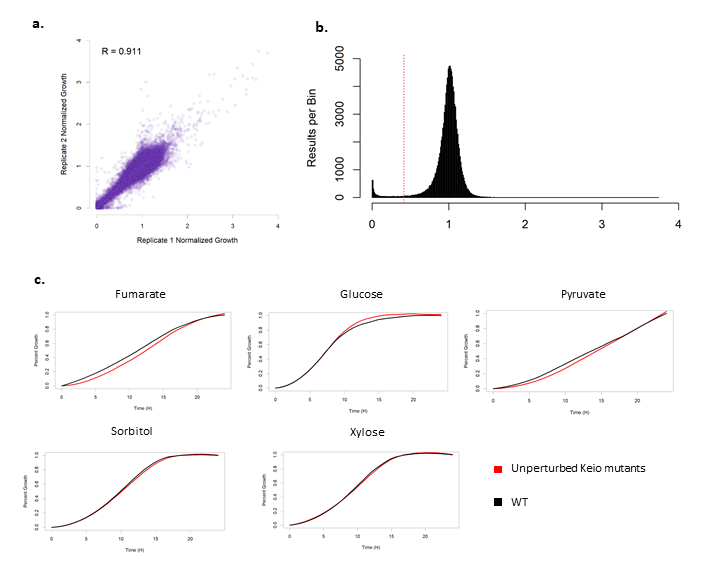

Supplement: FIG S3 [file mBio.02259-20-sf003.tif]

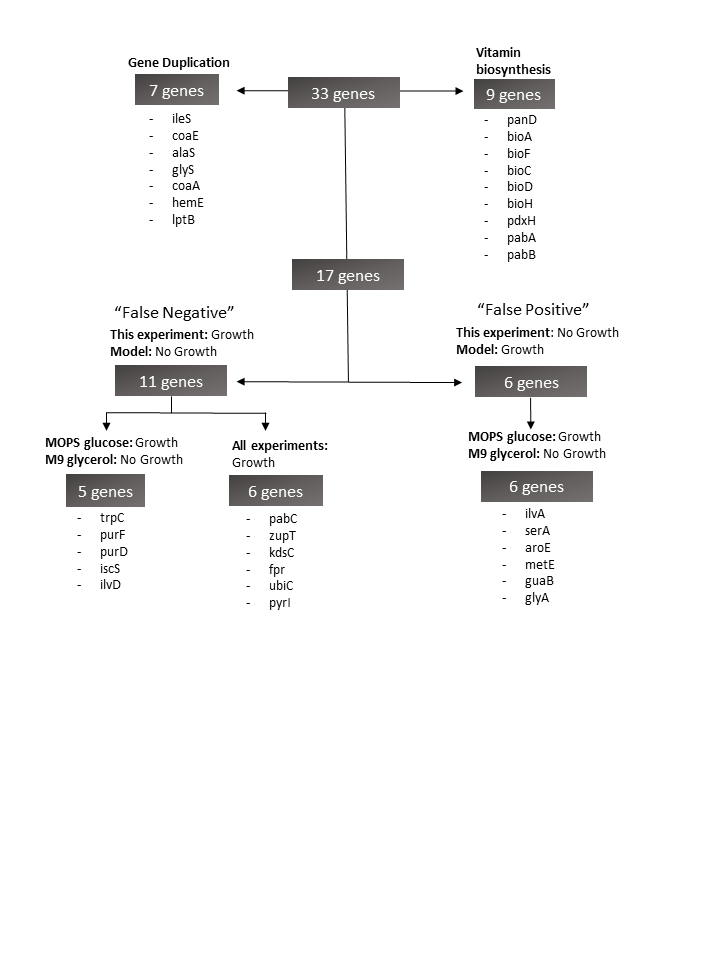

Supplement: FIG S4 [file mBio.02259-20-sf004.tif]
